# Multiple Venous Malformations as a Cause of Pulsatile Tinnitus

**DOI:** 10.1155/2020/8867963

**Published:** 2020-08-04

**Authors:** Ayham Al Afif, Uthman Alamoudi, Ahmed A. Al-Sayed, Manohar Bance

**Affiliations:** ^1^Division of Otolaryngology—Head and Neck Surgery, Dalhousie University, 1278 Tower Road, Queen Elizabeth II Health Sciences Centre, Dickson Building (3rd Floor), B3H 2Y9, Halifax, Nova Scotia, Canada; ^2^Department of Otolaryngology—Head and Neck Surgery, Hail University, Hail, Saudi Arabia; ^3^Department of Otolaryngology—Head and Neck Surgery, King Saud University, Riyadh, Saudi Arabia; ^4^University of Cambridge, Department of Clinical Neurosciences, Cambridge, UK

## Abstract

**Introduction:**

Pulsatile tinnitus is a relatively common presentation in otolaryngology clinics, most cases of which have a treatable cause. This presentation warrants a thorough workup to identify treatable, and rule out life-threatening, etiologies. We present a case of a patient with pulsatile tinnitus arising from multiple dilated venous channels in the head and neck. *Case Presentation.* We present the case of a 65-year-old Caucasian female with a two-year history of progressive, bilateral pulsatile tinnitus, which had become debilitating. Computed-tomographic angiography (CTA) studies ruled out an intracranial vascular cause for her symptoms. However, computed tomography (CT) scanning and magnetic resonance imaging (MRI) revealed multiple dilated bilateral, low-flow, venous channels throughout the head and neck. The proximity of such dilated venous channels to the temporal bone provides a route for sound to be transmitted to the inner ear.

**Conclusion:**

Arterial, venous, and systemic etiologies can cause pulsatile tinnitus. Arteriovenous malformations (AVMs) of the head and neck represent less than 1% of cases. In our patient, dilated low-flow venous malformations are the likely source of her symptoms, which is the first reported case in the literature.

## 1. Introduction

Tinnitus is defined as the perception of sound without external stimulus. It is divided broadly into 2 categories: nonpulsatile and pulsatile tinnitus. Studies have shown that the incidence of persistent tinnitus among the United States' population is 12–14% [1], with pulsatile tinnitus accounting for 4% of these cases. Despite how common pulsatile tinnitus is, its workup, diagnosis, and management pose a dilemma for the otolaryngologist [2]. Pulsatile tinnitus can arise from abnormal blood flow through dilated or stenotic vessel lumens within the head and neck, resulting in turbulent and audible blood flow. Alternatively, it can also stem from amplified bone conduction of normal physiological sounds within the inner ear, as seen in superior semicircular canal dehiscence (SSCD) syndrome. Pulsatile tinnitus can be classified as objective, if audible to the patient and examiner, or subjective if perceived by the patient only [3]. It can also be classified based on etiology, which can be venous or arterial. Common arterial causes include atherosclerotic carotid artery disease, intracranial arterial aneurysms, arteriovenous malformations (AVMs) or fistulas (AVFs), and tortuous internal carotid arteries [4]. Venous causes include abnormalities of the jugular bulb and sigmoid sinus and dilated mastoid emissary veins [5]. Idiopathic intracranial hypertension has been suggested as the most common cause of this presentation [6]. However, in a review and case-series by Hoffman et al., it was suggested that vessel-rich tumors of the head and neck are the most common cause of this presentation [7]. Systemic causes of pulsatile tinnitus include anemia and hyperthyroidism [8].

A comprehensive, and directed, workup is warranted for patients with pulsatile tinnitus, starting with a thorough history and physical examination. Careful otoscopic examination may reveal retrotympanic pathology, such as a glomus tumor. In patients with normal otoscopy, and a high risk of atherosclerotic arterial disease, duplex carotid ultrasonography should be attained to assess for carotid artery bruit as a possible etiology. When suspected, a lumbar puncture should be performed to rule out idiopathic intracranial hypertension. Sismanis et al. have highlighted that CT and CT-angiography (CTA) studies are most helpful in identifying arterial, and retrotympanic, causes of pulsatile tinnitus such as glomus tumors and AVMs or AVFs. Alternatively, MRI and MR angiography (MRA) studies are preferred in patients with a potential venous etiology [8–10].

## 2. Case Report

A 65-year-old female presented to our clinic with a 2-year history of worsening bilateral pulsatile tinnitus. She described “humming” in both ears, in time with her heartbeat, which had become very difficult to ignore, to the point of being debilitating. She had no history of hearing loss. She denied autophony, hearing her eyeballs move, dizziness on straining, or any other symptoms pointing to a diagnosis of SSCD syndrome. Her past medical history was significant for hypothyroidism, overactive bladder, hypertension, chronic back pain, and a remote history of thrombocytopenia. She also reported being diagnosed with multiple vascular lesions in the head and neck, and some of these had been surgically removed during childhood, including one superficial and posterior to the right ear. Her medications included levothyroxine, gabapentin, hydromorphone, oxybutynin, and omeprazole. A complete blood cell count was ordered, showing no anemia or thrombocytopenia. Audiometry showed no hearing loss.

Neurological and head-and-neck examination were normal. Her tympanic membranes appeared normal bilaterally. Pressurization of the neck bilaterally did not alleviate the intensity of the tinnitus. Her symptoms were ameliorated with rotation of the neck to the left. No murmurs or bruits were heard on auscultation.

**Figure 1:**
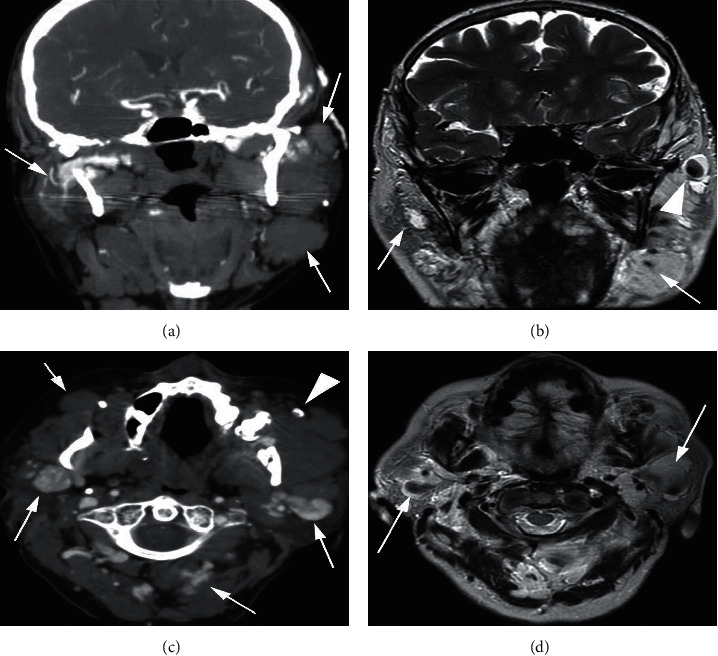
Contrast-enhanced CT ((a) coronal; (c) axial) and MRI (T2-weighted; (b) coronal; (d) axial). Note the numerous well-defined soft-tissue densities in the upper neck, which represent dilated, low-flow, venous channels (solid arrows, a–d). To avoid clutter, only some are pointed out. Calcified phleboliths are also notable within the dilated venous channels, which are commonly seen in low-flow venous malformations (arrow heads, b, c).

The patient underwent contrast-enhanced computed tomography (CT) and magnetic resonance imaging (MRI) scans (Figure 1) as part of the workup for her symptoms. The CT scan showed multiple, bilateral, enlarged, venous malformations in the neck and posterior to the ears (Figures 1(a) and 1(c)). The T2-weighted MRI (Figures 1(b) and 1(d)) scan showed similar findings. Notably, some of these dilated venous channels contained phleboliths, often seen in low-flow venous malformations. No intracranial or auricular malformations were seen. Magnetic resonance angiography (MRA) was also performed (not shown), revealing 2 small (<5 mm) incidental aneurysms in the paraophthalmic internal carotid artery, which were thought to not be causing any symptoms.

## 3. Discussion

Turbulent blood flow through vessels in the head and neck can be transmitted to the inner ear through surrounding bony and soft-tissue structures, contributing to the perception of pulsatile tinnitus [9]. In up to 30% of cases, no cause is identified. AVMs are a rare cause of pulsatile tinnitus. Hofmann et al. [7] showed that they contribute to less than 1% of published cases in the literature. The imaging characteristics seen in our patient show low-flow, dilated veins with phleboliths. Consequently, we believe that venous malformations rather than AVMs are the cause of tinnitus in this patient. To our knowledge, this is the first report of such a cause for pulsatile tinnitus.

Tinnitus arising from veins is often perceived unilaterally, relieved by ipsilateral venous pressure and accentuated by contralateral head turning. Focal venous malformations, such as dehiscent jugular bulbs and abnormal venous sinuses, contribute to less than 15% of cases [7]. Unique to our patient is the presence of multiple, bilateral, venous malformations in the head and neck, without any identifiable syndromes. The proximity of some dilated venous channels to the temporal bone (Figure 1(a)) provides a pathway for sound transmission to the inner ear [10]. Importantly, brain imaging alone might have missed many of these extracranial malformations.

Interestingly, low-flow, venous malformations have been associated with mutations in the *TEK* gene on chromosome 9p. This encodes the TIE2 tyrosine kinase receptor protein, which is important in smooth muscle cell recruitment and maturation. These mutations are also a common feature of several syndromes, including Klippel-Trénuanay and Maffucci [11]. Our patient lacked any distinct syndromic features and was not keen on genetic testing or interventions. We referred her to our audiology department of behavioral and suppressive therapy.

In conclusion, pulsatile tinnitus is a common presentation which warrants a thorough workup. The causes of this presentation are varied; venous, arterial, and systemic etiologies have been described. We present the case of a 65 year-old patient with progressive, bilateral pulsatile tinnitus. Imaging demonstrated multiple dilated, low-flow venous malformations in close proximity to the temporal bones. To our knowledge, this is the first case report of such presentation in the literature. Imaging, in the form of CT or MRI with or without angiography, is warranted in the workup of pulsatile tinnitus.
